# Gender bias in clinical research, pharmaceutical marketing, and the prescription of drugs

**DOI:** 10.3402/gha.v7.25484

**Published:** 2014-12-09

**Authors:** Elisa Chilet-Rosell

**Affiliations:** 1 Group of Public Health Research, University of Alicante, Alicante, Spain; 2 School of Medical Sciences, University of Cuenca, Cuenca, Ecuador

**Keywords:** clinical trials, gender bias, hormone replacement therapy, marketing, prescription, analgesia, gender development

## Abstract

This thesis is part of the studies of gender bias in health which together with the paradigm of evidence-based medicine shares the empirical assumption that there are inaccuracies in medical practice, in addition to a lack of rigour and transparency. It worked with the distinction between the concepts of sex and gender and between the concepts of sex-related differences and gender inequalities, in terms of applying a gender perspective in the study design and the subsequent analysis. This PhD review presents the research process conducted in Spain, which can provide an example for future research. Study I described a review of 58 clinical trials (CTs) of etoricoxib to assess its compliance with the Recommendations of Evaluation of Gender Differences in the Clinical Evaluation of Drugs. In Study II, key informants from professions related to different areas in drug development and pharmacovigilance held a working meeting to reach a consensus document on recommendations for the study and evaluation of gender differences in CTs in Spain. In Study III, the websites of the eight best-selling hormone replacement therapy drugs in Spain on Google first page of results were analysed. In Study IV, a logistic regression analysis was performed to compare analgesic prescription by sex in regions with a higher or lower Gender Development Index (GDI) than the Spanish average. Gender biases identified in this thesis limited the legitimacy of medicine, which is not based on the best possible evidence. The results also demonstrate the existence of inequalities between men and women that are not due merely to biological differences, but are gender inequalities stemming from the social differences that exist between both sexes.

This thesis is part of the studies of gender bias in health which together with the paradigm of evidence-based medicine shares the empirical assumption that there are inaccuracies in medical practice, in addition to a lack of rigour and transparency. This research was conducted in Spain, a country where inter-regional differences persist, showing a north–south pattern in gender development, pro-northern regions (1), even in recent years have reduced inequalities in development between women and men. In recent years in this country, there have been significant legislative changes in relation to equality between men and women in research.

The Organic Law 3/2007 of 22 March on effective equality between women and men (2) states that wherever possible, the data contained in records, surveys, statistics, and other medical information systems should be disaggregated by sex to facilitate a gender analysis. Point 3 of Article 27 states that through the health services and the competent bodies in each case and in accordance with the principle of equal opportunities, the public authorities will implement the following actions: Promotion of scientific research into differences between women and men related to health care, particularly as regards diagnostic and therapeutic accessibility and strategies, whether in clinical trials (CTs) or health care provision.

In the same vein, the Law 14/2011 of 1 June, on Science, Technology and Innovation (3), established the perspective of gender as a cross-sectional category in scientific and technological research which should be applied throughout all aspects of the process in order to guarantee effective equality between men and women.

## Gender bias

Making the presence of gender bias visible allows us to fill the gaps in knowledge about the health of women and men and to understand their health needs and risks, that is, to improve health care and interventions.

The *Journal of the American Medical Women's Association* defined gender bias in clinical practice in the 90s as ‘differences in the treatment of women and men with the same diagnosis, which may be positive, negative or neutral to the health of these’. The principal consequence is discrimination against one sex with respect to the other in the health services (4). The main problem with this definition is that it ascribes exclusive responsibility to health professionals. However, health professionals diagnose and treat according to the training received and the information available to them. Therefore, the real source of gender bias in clinical practice may be due to errors in research resulting from a lack of gender awareness. Then, and in line with the ideas of feminist empiricism, a more comprehensive definition of gender bias would be a systematic error related to a lack of gender awareness, leading to the mistaken view of men and women as similar (or different) in exposure to risks or in the natural history of a disease (symptoms and signs of onset and course, response to treatment and prognosis) (5, 6), and where the main consequence of gender bias in research and health care is the lack of valid results (6).

Thus, gender bias in health care is largely a result of gender bias in the generation of knowledge. However, we should also consider that marketing has become one of the most important filters of medical knowledge. Advertising in marketing campaigns can help to strengthen erroneously the perception that certain diseases are more frequent in one sex than another through greater representation of one of the two sexes. In addition, concern has been consistently expressed that pharmaceutical marketing contributes to the medicalisation of women's life processes (7).

**Fig. 1:**
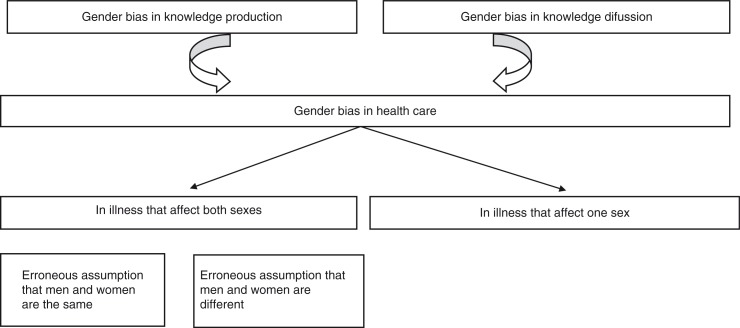
Interconnection between knowledge production, knowledge diffusion and health care.

A specific strategy has been employed for the research on which all the articles are based, namely, to identify gender bias. To this end, a working definition was used which was adapted to each context studied and from which the categories and variables to be analysed were selected. Focusing on therapeutic strategies, this PhD review comprises three interconnected contexts, including knowledge production, knowledge diffusion, and health care (Fig. 1).

## Gender bias in knowledge production

The erroneous assumption that men and women are the same is evidenced by the exclusive inclusion of men in CTs, with subsequent extrapolation of the results to women (5). The arguments in favour of the systematic exclusion of women from CTs have been based on foetal risk during pregnancy, hormonal interactions due to the menstrual cycle or the concomitant use of exogenous hormones [hormonal contraceptives and hormonal replacement therapy (HRT)], difficulties in recruiting, and higher dropout rates (8, 9). However, these reasons are precisely why women's participation in CTs is necessary.

Feminist empiricism postulates that sexism and androcentrism both constitute social biases which could be corrected through strict adherence to valid methodological standards in scientific research when testing hypotheses and interpreting data; in other words, within the context of the rationale. Feminism is proposed as a correction factor for already established theories. One of the strengths attributed to feminist empiricism is that it observes valid methodological standards in science, since it believes that the problem is bad science. The exclusion of women from CTs is an example of the poor application of the scientific method as a result of the androcentric nature of science. Under the false premise that men and women are the same, medications have traditionally been tested on men and the knowledge obtained about efficacy and effectiveness has been extrapolated to women.

**Fig. 2:**
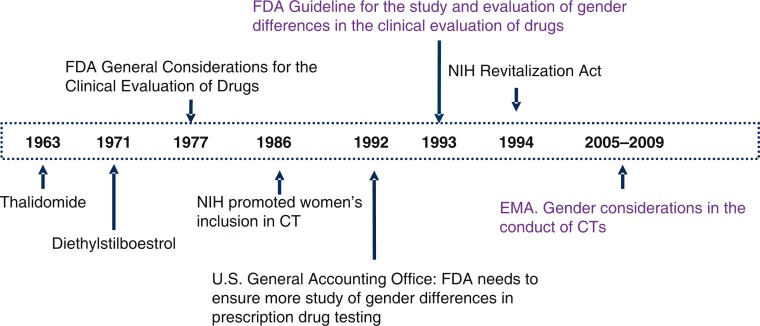
Summary of turning points in the regulation of women's participation in CTs.

Following the scandals related to the teratogenic effects of thalidomide and diethylstilboestrol (10), in 1977 the Food and Drug Administration (FDA) issued the ‘General Considerations for Clinical Evaluation of Drugs’ (11). This document recommended that women of childbearing age should not be included in the early phases of CTs, until sufficient data on drug toxicity had been obtained. In practice, this resulted in the exclusion of women from CTs (Fig. 2).

In the 1980s, the National Institutes of Health (NIH) began to promote women's inclusion in CTs (12); nevertheless, in 1992 the US General Accounting Office (GAO) voiced concern about the under-representation of women in CTs (13). In response, FDA published its ‘Guideline for the study and evaluation of gender differences in the clinical evaluation of drugs’, which argued that it was necessary to include women in CTs and also that the results should be stratified by sex and drug interactions with both endogenous and exogenous hormones being studied (14). Similarly, the NHI Revitalization Act was approved, making it a requirement that women be included in all NIH-funded CTs, or in the event that they are not then reasons for this be given (15).

The case in Europe is different. In 2005 and 2009, the European Medicines Agency (EMA) published ‘Gender considerations in the conduct of clinical trials’ (16), which stated that the publication of a specific guideline was not necessary. This statement was based on a review of the agency’s own data (about which little information is available) and using debatable arguments (17).

Since 1993, when the FDA lifted its ban on the inclusion of women in the early stages of CTs through the publication of its guidelines (14) and the NIH changed its policy (15) regarding the funding of clinical research, numerous studies have been published in scientific journals concerning the participation of women in CTs.

An editorial published in 1993 in the *European Journal of Public Health* highlighted the importance of re-examining results once they have been published, since if stratified by sex, the conclusions may be different for men and women (18). Therefore, literature reviews constitute an essential tool in gender studies. As part of this study, a literature review was conducted from a gender perspective on the CTs of a symptomatic drug with a questionable benefit–risk profile.

With the objective of contributing to the transfer of knowledge in order to support the incorporation of a gender perspective into the clinical research agenda in Spain, and more specifically into CTs, one aim of this PhD study was to develop a consensus document on the inclusion of women in CTs and the sex-stratified analysis of findings, in collaboration with a group of key informants in different areas of pharmacology and clinical research.

## Gender bias in knowledge dissemination

**Fig. 3:**
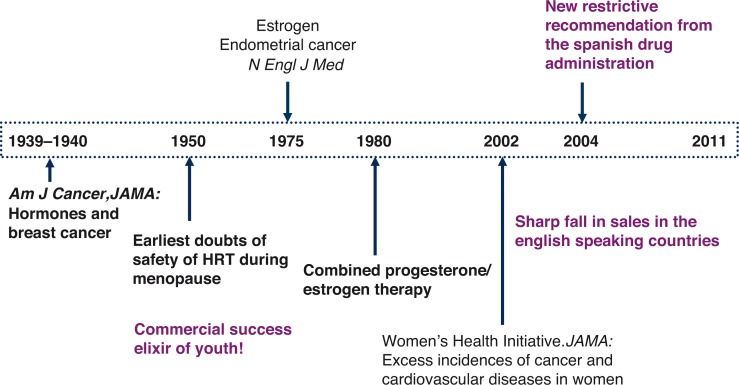
Summary of the history of HRT use and its relationship with adverse effects.

The feminist standpoint states that the dominant position of men in social life results in partial knowledge, whereas the subjugated position of women opens the possibility of a more complete knowledge (selection of research problems, definition of a problem, research priorities); in other words, within the context of discovery. So recognition of health problems specific to women has been slow, as is the case of HRT (Fig. 3). Towards the end of the 1930s, a debate arose about the relationship between oestrogen and breast cancer (19, 20), and during the 1950s, concern was voiced about its use during menopause (21). Nonetheless, HRT became a commercial success (22, 23). Following publication in the *New England Journal of Medicine* of the relationship between endometrial cancer and oestrogen (24, 25), combined HRT (oestrogen and progesterone) was released. Perhaps the most important event in the history of HRT was the publication of the results of the Women's Health Initiative (WHI) after the drug had been withdrawn due to the unacceptably high incidence of cancer and adverse cardiovascular effects in women taking HRT (26). Publication of the WHI results led to a sharp drop in sales in Anglo-Saxon countries. However, the Spanish Medicines Agency of Medicines and Health Products (SAMHP) waited until 2004 before restricting the indications for HRT (27), reaffirming these restrictions in 2008 (28). In fact, the biological, clinical, and epidemiological evidence highlighting the risks and refuting the alleged benefits associated with HRT has been a source of debate among pharmaceutical companies, epidemiologists, regulatory agencies, and feminist groups (29). The question posed by Nancy Krieger was ‘Why, for four decades, since the mid-1960s, were millions of women prescribed powerful pharmacological agents already shown, three decades earlier, to be carcinogenic?’ (29).

Despite the risks associated with HRT use, pharmaceutical marketing has presented the menopause, a natural part of women's life cycle, as pathological, creating the feeling of real illness among the population and ensuring that HRT is recommended and prescribed (30). The interests of certain medical specialities reluctant to stop prescribing hormonal drugs, particularly to women, together with those of the pharmaceutical industry and the media, which play such an important role in disseminating information to promote sales without considering the evident side effects (31), explain the importance of conducting research from a gender perspective which focuses on communication and advertising of hormonal drugs in Spain for commercial purposes. As Muir Gray aptly pointed out, it is a right and obligation of public health bodies to ensure that information is not misleading (32). In this respect, the European Union has voiced the need to review the rigour, integrity, and consistency of health information, including information available online (33), which has rapidly become the medium used by physicians and patients to access information on drugs (34) since it facilitates patient involvement by providing easily accessible information on health topics (35). For this reason, this PhD study also includes an analysis of the information about HRT drugs available online.

## Gender bias in health care

Historically, women's health has usually been seen almost exclusively in terms of reproductive health (36, 37). As a result of biological essentialism, knowledge about women's health is limited and does not take into account the ways in which the social reality of gender is manifested in women. Betty Friedan described how women's dissatisfaction was related to the fact that their opportunities were exclusively restricted to being a homemaker, wife, and mother (38). The emotional distress of women is medicalised by erroneous symptomatic treatment strategies which ignore the causes. Thus, it has been reported that women are more likely to be prescribed anti-anxiety drugs, sleeping pills, and medication for mental health problems than men (39). This may reflect a greater tendency among health professionals to attribute what are actually physical symptoms with atypical presentation to psychological factors when treating women, or a greater tendency to prescribe drugs for women than men when treating low-level depressive symptoms (40).

Although it has generally been observed that diagnosis and/or treatment are determined individually, they are nevertheless the result of a complex pattern of causes, including social determinants (41). When women enter the health care system, they may experience different treatment or diagnosis according to their status, social power, and socioeconomic status (42). In addition, it has been shown that women experience problems related to equality and quality of health care as regards access to specialists, one consequence of which is the increased prescription of symptomatic treatments (6, 43).

The discipline of epidemiology has shown no interest in investigating the reasons behind the contexts in which relationships between risk factors and disease arise (36). Due to the lack of information on the social determinants of health problems, the risks to women's health are often decontextualised and depoliticised (36). As with feminist empiricism, this critique suggests that there is an error in the application of scientific method, since important structural factors that affect men and women differently are not addressed.

Inequality between men and women has not traditionally been considered as a determinant of health care. It was Kawachi who published one of the first studies to indicate the importance of gender inequality as a social determinant of health, demonstrating the association between women's social status and morbidity in both sexes according to geographical region in the same country (44). A relationship has also been shown between women's empowerment and indicators of community health: women's empowerment and their participation in political, economic, and social life are associated with lower mortality are associated with child health and with lower mortality rates in men and women (45).

Consistent with a higher prevalence of pain, epidemiological studies have reported a greater use of analgesics among women than men. However, although logically, pain should be the main reason for prescribing analgesia, the literature indicates that pain alone does not explain higher rates of prescription to women, since given the same intensity of pain women are more likely than men to be prescribed analgesia (46). As Malterud has stated, doctors interpret symptoms differently according to whether they occur in a man or a woman (47). Thus, the prescription of analgesia depends less on the discomfort expressed by those experiencing it than on the perception that health professionals have of the patient in question (46, 48).

Given that individual factors do not explain the greater prescription of analgesia to women than men, and that contextual factors in health differences between men and women have not traditionally been studied, the present doctoral thesis includes an analysis of the influence of context, in this case gender development, in the prescription of analgesia in Spain.

The thesis worked with the distinction between the concepts of sex and gender and between the concepts of sex-related differences and gender inequalities, both in terms of applying a gender perspective in the study design and in the subsequent analysis. Below, we presented the research process which can be an example to future researches.

## Research process

### Gender bias in knowledge production: Study I (49) and Study II (50)

As part of this study, a literature review was conducted from a gender perspective on the CTs of a symptomatic drug with a questionable benefit–risk profile, namely, the anti-inflammatory, etoricoxib (Arcoxia, Merck Sharp & Dome) (51). This drug is mainly used by women, and the summary of product characteristics warns of interactions with female hormones (52). The 58 CTs reviewed were published between 2000 and 2007 to assess compliance of these CTs with the recommendations of the FDA guideline (14) and the Sex, Gender and Pain Special Interest Group Consensus Working Report (53). The protocol designed ad-hoc included these variables:

1. Sex differences (stratified data in order to enable gender analysis of the results, efficacy, adverse effects, dose–response, blood concentration–response and discussion of the results by sex).
2. Women-specific issues (pregnancy as an exclusion criterion, use of contraceptive methods, use of hormonal contraceptives, use of HRT, menstrual cycle and status).

A descriptive study of frequencies and percentages was performed for the aforementioned variables. After data collection, via email, we attempted to contact the 44 authors of the 54 papers that had published the 58 CTs on etoricoxib in order to discover any information about sex differences and gender information that had not been published in their papers.

To develop a consensus document on the inclusion of women in CTs and the sex-stratified analysis of findings, this study had the collaboration of a group of key informants in different areas of pharmacology and clinical research (Ethics Committees for Clinical Research, Universities, Health Service CT inspection, pharmacovigilance, CT research and development companies, the SAMHP and the EMA, providing added transfer value to the research). First, key informants were provided with the relevant literature on women's inclusion in CTs, evaluation of gender differences, and sex-stratified analyses of CTs of a new drug (3–8, 15, 16), and they responded individually to an open-ended questionnaire about the challenges of conducting CTs to determine gender differences in pharmacokinetics, the problems arising from the failure to consider these differences in trials, and the establishment of priorities according to the feasibility of systematically incorporating the ‘gender difference’ factor in each phase of the development of a new drug. Once the information given in the responses had been processed, a working meeting was held with key informants and participants; then a debate was held in order to reach consensus on the suitability of developing recommendations for studying and evaluating gender differences in CTs in Spain, the content of such recommendations, and the steps to take in order to improve information on gender differences and implement gender analyses in the process of developing a new drug.

### Gender bias in knowledge diffusion: Study III (54)

A search was carried out on the Internet (January 2009) using the eight best-selling HRT drugs in Spain (information obtained from the Spanish Ministry of Health and Consumer Affairs General). The brand name of each drug was entered into Google's search engine. The websites appearing on the first page of results and the corresponding companies were analysed using the European Code of Good Practice as the reference point:

1. Restriction of the information to a specialised public.
2. Obligatory inclusion of the drug's summary of product characteristics.
3. Conformity of the indications with the patient information leaflet.
4. Inclusion of the registered company name and address.
5. Inclusion of complete bibliographic references, including references to the original source.

Additionally, in non-corporate web pages, information was collected about whether the page offered links to sites selling medicines and erroneous or inadequate information concerning drug indications. In the case of online pharmacies, the possibility of purchasing prescription-only medication from Spain was also studied.

### Gender bias in health care: Study IV (55)

A cross-sectional study of sex differences in analgesic prescription according to the gender development of the regions studied was performed. Analgesic prescription, pain, and demographic variables were obtained from the Spanish Health Interview Survey in 2006. Gender development was measured with the Gender Development Index (GDI). For this study, we used a dichotomous classification of the 2005 GDI for the different regions in Spain; below the national average in Spain or above, based on previous work (1). This classification showed a north–south pattern whereby gender development in northern regions was above the national average whilst in the south it was lower. A logistic regression analysis was conducted to compare analgesic prescription by sex in regions with a GDI above or below the Spanish average, adjusted for pain confirmed by a doctor, age, and social class.

## Results

### Gender bias in knowledge production

In etoricoxib CTs reviewed, 70% of the 49,835 subjects were women. However, if we disaggregate data by phase of CTs, only 30% of the participants were women. Nearly 85.7% of the trials did not stratify by sex. With regard to efficacy, 90.6% did not perform an analysis by sex, and this figure rose to 93.3% in the case of adverse effects. Little information is available on the possible interactions of exogenous and endogenous hormones with the drug. The authors interviewed by our e-mail query indicate arguments related to insufficient sample size to detect such differences. Another answer was that no sex analysis had been carried out, but that subsequent studies on the need for analgesics during the post-operative phase had detected sex differences.

According to the answers from the experts, women should be included in the development plan of any drug that they may potentially use and should be included in CTs from the early stages of drug development. Additionally, sex-disaggregated results should be given in CTs as regards efficacy, adverse effects, dose response, and blood concentration. Studies should be conducted on the possible interactions of both endogenous and exogenous hormones with drugs. In accordance with the Spanish Law on Equality, the experts considered that a consensus document should be created that systematically supports compliance with the necessary requirements throughout all stages of the development plan of a new drug, including publication of results.

### Gender bias in knowledge diffusion

In the case of the best-selling HRT drugs in Spain, of the five pharmaceutical company websites analysed, none gave bibliographical references or included measures to ensure that the information was only available to health professionals. Of the 27 non-corporate websites, 41% did not give the company or registered name, 44% made no distinction between patient and health professional information, and only 7% gave bibliographical references. Among the indications given, 26% included use for the prevention of osteoporosis and 19% included regulation of the menstrual cycle, and even to enhance femininity. Two online pharmacies were identified that sold HRT, available for purchase from Spain. Their sites did not give the name or contact details of the company or indicate that a medical prescription is required for HRT.

### Gender bias in health care

Independent of pain, age, and social class, women were more likely to be prescribed analgesia than men OR=1.74 (1.59–1.91), as were residents of Autonomous regions with lower GDIs compared to residents of regions with higher GDIs: OR women=1.26 (1.12–1.42), OR men=1.30 (1.13–1.50). Women with pain in Autonomous regions with a lower GDI were more likely to be treated by a general practitioner rather than a specialist, OR=1.32 (1.04–1.67).

## Discussion

Although there is a growing number of policies, at least in Spain (2), aimed both at increasing women's presence in the population forming part of a CT and encouraging analysis of the results by sex, women are still under-represented in CTs. The first of the articles identified gender bias in the context of rationale, based on the under-representation of women in CTs of etoricoxib, particularly in the initial phases. These initial phases are important for detecting sex-related differences regarding pharmacokinetics and pharmacodynamics and for providing the information that is used to design subsequent CT phases. The other important finding was the virtual absence of any analysis by sex of the results obtained in the etoricoxib CTs. The under-representation of women in the design phase of CTs is probably the reason behind the failure to analyse the results by sex or to consider the interaction of women's hormonal changes with the drug. As suggested by feminist empiricism, inadequate methodological design due to lack of gender awareness in the context of clinical research has been a determining factor in the emergence of concerns about the validity of the knowledge produced about this drug (Table 1). This is particularly relevant given that etoricoxib, with its controversial risk–benefit profile, is widely prescribed to and taken by women with musculoskeletal problems, which are common as we get older and affect women more than men as the former have a longer life expectancy. The study indicated that etoricoxib may pose the same problems for women as other drugs in its class (COXIBs).

**Table 1:** Summary of the methodological design of the doctoral thesis

	Study I	Study II	Study III	Study IV
Title	Women's health and gender-based clinical trials on etoricoxib: methodological gender bias	Recommendations for the study and evaluation of gender differences in clinical trials in Spain	Hormone replacement therapy advertising: sense and nonsense on the web pages of the best-selling pharmaceuticals in Spain	Inequality in analgesic prescription in Spain. A gender development issue
Objective	To determine compliance with published good practice guidelines for gender and clinical trials using etoricoxib	To analyse the appropriateness and quality of Internet advertising about HRT in Spain	To develop a consensus document on the inclusion of women in CTs and the sex-stratified analysis of findings, in collaboration with a group of key informants in different areas of pharmacology and clinical research	To analyse factors associated with analgesic prescription according to levels of gender development in Spain, considering pain, age, and social class
Feminist critiques of science	Feminist empiricism		Feminist standpoint theory	Feminist empiricism
Gender bias	Erroneous assumption in CTs that men and women are the same		Medicalisation of life processes	Erroneous assumption that men and women are different with respect to a health need
Observation context	Production of knowledge		Knowledge diffusion	Health care
Methodology	Literature review of published CTs Protocol: FDA guideline for the study and evaluation of gender differences in the clinical evaluation of drugs (14) and the Sex, Gender and Pain Special Interest Group Consensus Working Group Report (53) Descriptive	Study using key informants Ad hoc open-ended questionnaire and meeting with key informants Descriptive	Study of the content of websites promoting the best-selling HRT in Spain. Protocol: European Code of Good Practice for the Promotion of Medicines Descriptive	Cross-sectional study Variables of interest taken from the Spanish National Health Survey (2006) Gender Development Index (GDI) of the Autonomous regions in Spain Multivariate logistic regression analysis
Main conclusion	Women are under-represented in, specifically, Phase I. Sex-stratified data on efficacy and adverse effects are scarce. Lack of data on women-specific issues. Etoricoxib may pose the same potential problems for women as other cyclooxygenase-2 inhibitors.	Women should be included in the development plan of any drug that they may potentially use, from the early stages of drug development. Sex-disaggregated results should be given in CTs. Studies should be conducted on the possible interactions of hormones (endogenous or exogenous)	Deficiencies were observed regarding the identification, information and promotion of HRT drugs on their web pages. Non-corporate web pages are an ideal place for indirect HRT advertising, but they often contain misleading information. HRT can be bought online from Spain, without a medical consultation or prescription	Once adjusted by pain, age and social class, women were more likely to be prescribed analgesics than men, as residents in regions with a lower GDI Women experiencing pain in regions with a lower GDI were more likely than men to be treated by a general practitioner rather than by a specialist irrespective of age and social class.

One of the main arguments put forward by pharmaceutical companies for not conducting an analysis by sex or an analysis of hormonal interactions is the cost involved in using a sufficient sample size to detect differences. However, withdrawing a drug from the market due to the higher presence of adverse effects in women, possibly related to lack of information about this part of the population, is even more expensive (56). Furthermore, economic cost is an argument which loses its impact when one realises that companies spend more on marketing than on research and development (57). In opposition to the arguments used by pharmaceutical companies and agencies such as the EMA, which do not consider the inclusion of women in CTs or the study of sex-related differences to be relevant, we must continue to insist on improving the findings of this ‘evidence-based’ medicine through the implementation in Europe of recommendations such as those of the FDA, until the pharmaceutical companies and the EMA come to accept the added value of incorporating sex–gender analyses in clinical research, for their authority and their own interests, including commercial interests.

As mentioned earlier, one of the largest proportions of a pharmaceutical company's budget is spent on developing marketing strategies to encourage the consumption of drugs. For decades, the use of HRT has been widely debated in terms of the risks associated with its consumption and the health benefits for women. However, using the new information technologies and taking advantage of the limited legislative control, companies recommend HRT to prevent osteoporosis (where the increased risk of bone fractures is described as a disease) and other indications which could entail long-term consumption, ignoring the restrictions on HRT use approved by the SAMHP. Furthermore, the information found on their websites is confusing and does not adhere to existing regulations. On the websites analysed, included were uses which had not been approved by the health authorities and it was also easy to buy HRT. Such a situation is extremely serious when considering that HRT is a potent drug with thrombogenic and carcinogenic effects (26), and that patients increasingly turn to the Internet to find information about health and medication (34).

As proposed by the feminist standpoint critique, in the case of HRT, pharmaceutical marketing does not give sufficient weight to the risks that may affect women's health. Thus, pharmaceutical marketing can be detrimental to the health of women, by reinforcing gender stereotypes in the social construction of disease and thereby contributing to the generation of gender bias in health care (58–60).

It is precisely for this reason that the study of health care, which forms part of this study, has focused on the prescription of analgesia from a gender perspective. Gender is one of the fundamental determinants of health inequalities, according to the Spanish Commission on Social Determinants of Health Inequalities (61). This has been demonstrated by ample evidence indicating that compared with men, women have unequal access to health resources, and even more so to quality resources (6, 43, 62). Thus, patterns of disease and treatment reflect the political and economic characteristics of society and social inequalities (44, 45, 63, 64). This study adds a contextual factor to the analysis of inequalities in prescribing analgesia, showing that the political and economic characteristics of society influence health and treatment of health problems: being a woman and living in areas with a lower GDI constitute two conditions that increase the likelihood of analgesia prescription, and the latter condition also affects men. Furthermore, the results show that women, especially those living in an area with a lower GDI, receive symptomatic treatment of pain more often than men, which could indicate that they do not receive adequate health care with regard to the underlying cause of their symptoms. The scientific literature indicates that women may encounter more obstacles to accessing specialised services than men. The consequence is that the potential conditions that may be causing pain are disregarded, reducing the possibility of benefiting from the prescription of appropriate treatment (43, 65).

## Limitations and strengths

There are some limitations related to the studies of this PhD thesis. In first place, it is not possible to guarantee that all available clinical information on etoricoxib has been reviewed; however, the review of the 58 CTs analysed provides new empirical evidence consistent with other publications questioning the validity of the information for both sexes. Consultation with the authors of the CTs analysed regarding their reasons for not including an analysis of sex-related differences in the CTs confirmed the relevance of performing these analyses since the authors themselves were in favour.

The key informant technique presents certain drawbacks, but one of the strengths of our study is that it included professionals from different areas of pharmacology and clinical research, even though pharmaceutical companies did not participate.

Due to the dynamic nature of the Internet, the results for websites promoting HRT may vary when searching on a different date to that when the original search was conducted (January 2009). Thus, the review of information from the websites retrieved on the first page of Google results does not include all the information available on the Internet about each of the drugs analysed. The Internet literature search on HRT drugs simulated the kind of search any HRT user might carry out, since increasing number of patients now seek information about diseases and drugs online.

Although it has been shown that the Spanish National Health Survey is a valid instrument which is widely used to describe patterns of drug prescription, the questionnaire presented some limitations that affected the study of analgesia prescription discussed in this thesis. However, the application of a gender perspective in the analysis of analgesia prescription patterns added a contextual factor that conditions the treatment of pain, differentiating it from individual risk and therefore offering a dual, more comprehensive vision of reality.

## Conclusions

The results extracted from the articles indicate gender bias in the production of knowledge about drugs, in the dissemination and marketing of these, and in therapeutic strategies in professional practice. The gender biases identified included extrapolation to the general population of information obtained from CTs which did not reflect the proportion of women who would consume the medication and which did not take possible hormonal interactions into account. These results indicate unawareness from agencies involved in drug development. In Spain, Spanish Agency for Medicines and Health Products should encourage compliance with the Organic Law 3/2007 for effective equality between women and men. Drug advertising could represent a serious public health problem, as it is characterised by containing errors that reinforce and/or increase the gender biases identified in CTs, though not including information on risks to women and extending the therapeutic indications for HRT, and this presents a serious public health problem. Finally, the findings for professional practice revealed the existence of gender bias in therapeutic strategies. Excessive levels of symptomatic treatment were identified in terms of analgesic prescription to women and in regions with a lower GDI. Gender bias may be one way in which inequalities in analgesic treatment adversely affect the health of women, through the medicalisation of women's discomfort.

This limits the legitimacy of medicine, which is not based on the best possible evidence. The results also demonstrate the existence of inequalities between men and women, which are not due merely to biological differences but are gender inequalities stemming from the social differences that exist between both sexes. Gender based medicine expects that the results of research strengthen the validity of medicine.

### What's next?

The generation and dissemination of knowledge biased from a gender, the perspective and its subsequent implementation, both in health practice and health policy can continue to perpetuate inequalities in the health of women and men. Therefore, it is necessary to delve into the factors involved in social inequalities between men and women to provide answers about the causes of health inequalities and, consequently, the key to its disappearance. Gender analysis is to figure out how sex–gender system impacts the health of women and men, thus generating health inequalities.

This thesis worked with different strategies depending on the context, defining working definitions of gender bias, and applying the gender perspective in the design and analysis of research, thus may serve as a benchmark for future research.

It is crucially important to identify gender bias and understand how it operates in medicine, as is socially harmful and expensive; there is a growing demand for information from the gender feminist movements, international conferences and forums, college programs in the area of gender, and the requirements of international agencies sponsoring projects. But a new perspective has emerged, it is called Gendered Innovations, that harnesses the creative power of sex and gender analysis to discover new things, by integrating methods of sex and gender analysis into basic and applied research, Gendered innovations produces excellence in medicine (66). The knowledge generated by both perspectives, gender bias and gendered innovations, can be a powerful tool that contributes to the gender-based medicine.
